# Species delimitation of Chinese hop‐hornbeams based on molecular and morphological evidence

**DOI:** 10.1002/ece3.2251

**Published:** 2016-06-13

**Authors:** Zhiqiang Lu, Dan Zhang, Siyu Liu, Xiaoyue Yang, Xue Liu, Jianquan Liu

**Affiliations:** ^1^ State Key Laboratory of Grassland Agro‐Ecosystem College of Life Science Lanzhou University Lanzhou China; ^2^ MOE Key Laboratory for Bio‐resources and Eco‐environment College of Life Science Sichuan University Chengdu China

**Keywords:** Genetic gaps, incomplete lineage sorting, internal transcribed spacer, introgression, *Ostrya*, species delimitation

## Abstract

Species delimitation through which infers species boundaries is emerging as a major work in modern systematics. Hop‐hornbeam species in *Ostrya* (Betulaceae) are well known for their hard and heavy woods. Five species were described in China and their interspecific delimitations remain unclear. In this study, we firstly explored their distributions in all recorded field sites distributed in China. We then selected 110 samples from 22 natural populations of five species from this genus and one type specimen of *O. yunnanensis*, for molecular barcoding analyses. We sequenced four chloroplast (cp) DNA fragments (*trnH–psbA*,* trnL–trnF*,* rps16,* and *trnG*) and the nuclear internal transcribed spacer (ITS) region for all samples. Sequence variations of *Ostrya* from four cpDNA fragments identified three groups that showed no correspondence to any morphological delimitation because of the incomplete lineage sorting and/or possible interspecific introgression in the history. However, phylogenetic analyses of ITS sequence variations discerned four species, *O. japonica, O. rehderiana, O. trichocarpa,* and *O. multinervis* while *O. yunnanensis* nested within *O. multinervis*. Morphological clustering also discerned four species and showed the complete consistency with molecular evidence. Moreover, our phylogenetic analyses‐based ITS sequence variations suggested that *O. trichocarpa* comprised an isolated lineage different from the other Eurasian ones. Based on these results, hop‐hornbeams in China should be treated as four separate species. Our results further highlight the importance of ITS sequence variations in delimitating and discerning the closely related species in plants.

## Introduction

Species delimitation through which species boundaries are determined (Sites and Marshall 2003) is becoming popular (De Queiroz 2007; Tavares and Baker 2008; Yang and Rannala 2010) and may be an important work in modern systematics, especially based on molecular evidence at the population level (Ross and Shoemaker 2005; Leaché and Fujita 2010; Fujita et al. 2012). As a basic and important unit for biological classification (Mayr 1963; McKenna and Bell 1997), species is the cornerstone in all classification‐based researches (Wiens 2007). Any invalid species, especially those listed as endangered and precious ones, may waste financial resources in their unnecessary protections (Murphy 1989; Rojas 1992). In addition to statistical clustering of morphological traits at population level (e.g., Snijman 1997; Türkoğlu et al. 2003; Duminil and Di Michele 2009; Yu et al. 2010), genetic gaps based on molecular evidence provide an independent and objective criterion for species delimitation. Undoubtedly, this operational approach may be convenient and practical. The sequence variations, especially based on DNA barcodes, should be firstly considered for this aim for two reasons. First, these DNA barcodes were designed to discern the extant species and genetic gaps provided by them in turn should be treated as a criterion to delimitate species boundary (e.g., Chase et al. 2005; Kress et al. 2005). In doing so, the delimitated species can be further discerned in the future by these DNA barcodes (Hu et al. 2015; Su et al. 2015). Second, all DNA barcodes are easy and cheap to be amplified and sequenced for multiple individuals (e.g., Wang et al. 2011b). Therefore, genetic gaps between morphological clusters can be easily determined.

In plants, the suggested DNA barcodes comprise the chloroplast (cp) DNA fragments (i.e., *rbcL*,* matK*,* trnH–psbA,* and *trnL–trnF*) (Chase et al. 2005; Kress et al. 2005; Kress and Erickson 2007; Fazekas et al. 2008; CBOL Plant Working Group 2009; Hollingsworth et al. 2011) and the nuclear internal transcribed spacer (ITS) region (Li et al. 2011; Pang et al. 2011; Wang et al. 2011b). Other cpDNA fragments such as *rps16* and *trnG* with high mutation rates are occasionally used (Shaw et al. 2007; Dong et al. 2012). However, the discrimination power of these barcodes, especially between nuclear ITS and cpDNAs, varies greatly depending on the studied groups (Petit and Excoffier 2009; Zhou et al. 2010; Wang et al. 2011a; Hu et al. 2015; Su et al. 2015). At most scenarios, ITS sequence variations were found to be effective in discriminating the closely related species with the relatively recent divergences (Li et al. 2011; Wang et al. 2011b; Su et al. 2015).

In this study, we used these DNA barcodes to delimitate species boundaries of the genus *Ostrya* Scopoli (Betulaceae) in China. All species of this small genus, called as hop‐hornbeams, are famous for hard and heavy woods. Five species were recorded in China (Li and Skvortsov 1999; http://www.efloras.org). *Ostrya japonica* Sargent, the type species of the genus, is widely distributed from eastern to northwest China while another species *O. multinervis* Rehder was sparsely recorded in southern and southwest China (http://www.cvh.ac.cn/). The remaining three species seem to be narrowly distributed. One famous endangered species, *O. rehderiana* Chun, was reported to have only retained five mature trees in Tianmu Mountains in Linan, Zhejiang (Ren et al. 2012), although more offspring individuals from these maternal trees were cultivated. No more distribution was reported except for the only locality of the type specimens for *O. yunnanensis* Hu ex P. C. Li. This species was suggested to be conspecific to *O. multinervis* by some authors due to their overlapped morphological traits (Wu 1991). Additionally, taxonomic position of *O. trichocarpa* D. Fang and Y. S. Wang needs to be reconsidered through molecular evidence because few specimens were collected for this species and the morphological characteristics are not enough to warrant its species status (Fang and Wang 1983). Previous phylogenetic studies on this genus were involved in a few species as well as a few individuals of the sampled species (Yoo and Wen 2002, 2007; Li 2008; Grimm and Renner 2013). Hence, the major aims in this study were to address the following questions: (1) How many species should be delimitated in Chinese hop‐hornbeams based on molecular and morphological evidence at the population level? What is the interspecific relationship for the newly delimitated species based on the newly available data? (2) Which barcode among the nuclear ITS region and cpDNA fragments is the most effective in delimitating and discerning the closely related species?

## Materials and Methods

### Field exploration and sample collection

We firstly investigated all populations in the field according to the specimen records of the genus *Ostrya*. Most populations of widely distributed species, *O. japonica* can be found in the field and we collected 14 populations across its distribution ranges. However, we found that no more extant tree was found for some populations of *O. multinervis* according to the specimen records. We only found three populations in southern China and one in southwest China. We collected samples from all of five extant large and mature trees of *O. rehderiana* in Linan, Zhejiang. We did not sample young trees or seedlings because they were artificially cultivated by seeds from these five large trees. However, seven specimens of mature offspring from the five extant tree of *O. rehderiana* were collected for morphological measurement. The type specimen of *O. yunnanensis* was collected from Luquan, Yunnan. We explored the type location and found only one tree left. Except for a sample from this tree, we further collected the fragmented leaves on the type specimen of this species for analyses. We explored the field where the specimens of *O. trichocarpa* were collected in Guangxi and Guizhou. Finally, we acquired two populations. In addition, for most natural populations, we strictly followed the principles that the trees from each population of each species we collected its leaves must be spaced at least 50 meters apart. We immediately used the silica gel to dry the fresh leaves. All representative specimens were deposited in the Lanzhou University. The elevation, latitude, and longitude of every location were measured and recorded using a handheld GPS unit. The details about the sample collection are in Table 1. In total, we used 112 samples for our molecular analyses containing 22 natural populations and the fragmented leaves from the type specimen of *O. yunnanensis*.

**Table 1:** Location of populations, number of individuals used in internal transcribed spacer (ITS) and cpDNA haplotype distribution per population of five species in *Ostrya*

Population code	Location	Latitude (N)	Longitude (E)	Altitude (m)	*N*_1_	*N*_2_	Haplotypes (no. of individuals)
*O. japonica*							
MZL	Muzaling, Henan	33°44′	112°15′	1620	5	5	C1 (5)
HS	Huoshan, Anhui	31°08′	116°11′	985	5	7	C4 (7)
QS	Qinshui, Shanxi	35°26′	112°01′	1589	5	5	C1 (5)
LG	Langao, Shaanxi	32°08′	108°48′	1452	4	7	C1 (5)/C2 (2)
ZQ	Zhouqu, Gansu	33°35′	104°21′	1772	4	6	C2 (6)
PW	Pingwu, Sichuan	32°38′	104°31′	1410	6	6	C7 (2)/C8 (4)
TS	Tianshui, Gansu	34°19′	106°12′	1526	3	3	C1 (1)/C2 (2)
SB	Songbai, Hubei	31°47′	106°12′	1420	1	1	C2 (1)
HP	Hongping, Hubei	31°34′	110°26′	1718	5	5	C1 (3)/C2 (2)
LC	Luanchuan, Henan	33°48′	111°20′	1386	4	6	C1 (5)/C2 (1)
LS	Lushi, Henan	33°45′	110°50′	1534	5	8	C1 (8)
MX	Meixian, Shaanxi	34°02′	107°53′	1540	1	1	C2 (1)
LX	Lixian, Sichuan	31°24′	102°59′	2380	3	3	C1 (3)
NS	Ningshan, Shaanxi	33°34′	108°29′	1638	3	3	C2 (2)/C5 (1)
*O. multinervis*							
WC	Wencheng, Zhejiang	27°50′	119°48′	610	6	12	C3 (12)
LS	Lishui, Zhejiang	27°54′	119°12′	800	1	1	C3 (1)
XN	Xinning, Hunan	26°36′	111°06′	1260	1	1	C1 (1)
YJ	Yinjiang, Guizhou	27°55′	108°38′	1253	6	12	C1 (12)
*O. yunnanensis*							
LQ	Luquan, Yunnan	26°08′	102°41′	2392	1	1	C1 (1)
*O. rehderiana*							
LA	Linan, Zhejiang	30°19′	119°27′	370	5	5	C4 (5)
*O. trichocarpa*							
LB	Libo, Guizhou	25°18′	107°56′	956	5	5	C6
NP	Napo, Guangxi	23°15′	105°35′	1260	7	7	C9
Total					86	110	

*N*_1_, number of individuals analyzed for ITS; *N*_2_, number of individuals analyzed for cpDNA data.

### DNA isolation, amplification, sequencing, and clone experiment

The total DNA of all samples was isolated from approximately 20 mg dried leaves according to the modified cetyl trimethyl ammonium bromide (CTAB) procedure (Doyle and Doyle 1990). We used primers of seven cpDNA fragments (Table S1) to amplify and sequence 16 individuals collected from 16 locations for four *Ostrya* species (*O. trichocarpa* was excluded) in China. We failed to detect sequence variation between these individuals for three cpDNA fragments: *trn*V–*trn*M, *mat*K, and *rbc*L. We therefore used the remaining four cpDNA fragments, *psbA*–*trnH*,* trnL*–*trnF*,* trnG* ^(UCC)^ intron, and *rps*16 for all samples.

We performed the PCR amplification in a 25 *μ*L volume containing 1 *μ*L of plant DNA with 50–100 ng/*μ*L, 2.5 *μ*L 10 **× **PCR buffer, 0.5 mmol/L of dNTPs, 2 *μ*mol/L of each primer, 0.2–0.3 *μ*L rTaq polymerase (5 U/*μ*L; TaKaRa, Dalian, China), and the ddH_2_O added to 25 *μ*L. The cycling parameters were coded for an initial denaturation step at 94°C for 4 min, followed by 38 cycles of 40 sec at 94°C, 45 sec at 54°C (*psbA–trnH*) or 59°C (*trnL–trnF*,* rps*16) or 58°C (*trnG*), 1 min and 20 sec at 72°C, and the final extension step is 7 min at 72°C. All PCR products were examined through the agarose gel electrophoresis. Then, the qualified products were purified using a TIANquick Midi Purification Kit according to the protocol (TIANGEN, Beijing, China).Subsequently, the sequencing reactions were performed with either or both primers and we sequenced the reaction mixtures using an ABI 3130xl automated sequencer (Applied Biosystems, Foster City, CA).

For the isolated DNA from the type specimen of *O. yunnanensis*, we tried to use Primer STAR HS DNA Polymerase (Takara, Dalian, China) to amplify the ITS, *psbA*–*trnH*,* trnL*–*trnF*,* trnG,* and *rps*16 genes. However, we failed in the amplification of this specimen using this method. We repeated the amplification for them using the MightyAmp polymerase according to the specific protocols and only ITS fragment was amplified successfully. Subsequently, we found all the directly sequenced ITS productions showed heterozygotes. These PCR products were further mixed and cloned into the pMD18‐T Vector (Takara, Dalian, China) after being purified using a TIANGEN Purification Kit. A total of 30 clones were randomly selected and cultured by isolated plasmids. We further screened clones through PCR amplification and gel electrophoresis, and the qualified positive clones were chosen to be sequenced using universal primers (Table S1).

We obtained 440 cpDNA sequences for 110 individuals for final analyses. We only selected a few individuals from the same population with different cpDNA haplotypes for sequencing ITS fragment. In total, we obtained 86 ITS sequences for the final analyses.

### Phylogenetic analyses

Aligning the sequences from ITS region and cpDNAs was performed by the software MAGA v5.0 (Tamura et al. 2011). We converted the data format using ClustalX v2.11 (Thompson et al. 1997). Phylogenetic analysis of the aligned cpDNA haplotypes and ITS sequences was performed by maximum parsimony (MP) using the software PAUP* v4.0b10 (Swofford 2002). All indels treated as single mutation events were coded as the fifth status. MP analysis was performed using heuristic search parameters which were simple additions of sequences of taxa combined with MULPARS and TBR (tree bisection–reconnection) branch‐swapping, ACCTRAN optimization, and 1000 random addition replications for the two datasets. Bootstrap values were calculated with 1000 replications of the heuristic search with simple taxon addition and TBR and MULPARS options selected in PAUP* (Swofford 2002). *Ostryopsis nobilis* I. B. Balfour and W. W. Smith was defined as an outgroup species. We also used median‐joining networks with NETWORK v4.6.1.1 (Bandelt et al. 1999; available at http://www.fluxus-engineering.com) to construct the interspecific relationships among cpDNA haplotypes.

### Morphological measurements of the variable traits

We reexamined morphological characters of all five *Ostrya* species based on specimens including bract, nutlet, and leaves (Li and Skvortsov 1999). We measured all traits which were assumed to differentiate five species (Li and Skvortsov 1999). We selected seven quantitative and three qualitative traits that were found to be obviously variable between and within species (Table S2). We selected 2–10 typical specimens for each population for measurements. In total, 135 individuals from 22 natural populations were used for statistical analyses. We also compared the morphological differentiation between *O. yunnanensis* specimens collected from the same tree at different years showing the difference in the leaf hair coverage. In order to visually and clearly demonstrate the variations among species through the statistical data, we produced a graphical representation.

## Results

### Sequence variation at four cpDNA fragments and phylogenetic analyses

**Figure 1:**
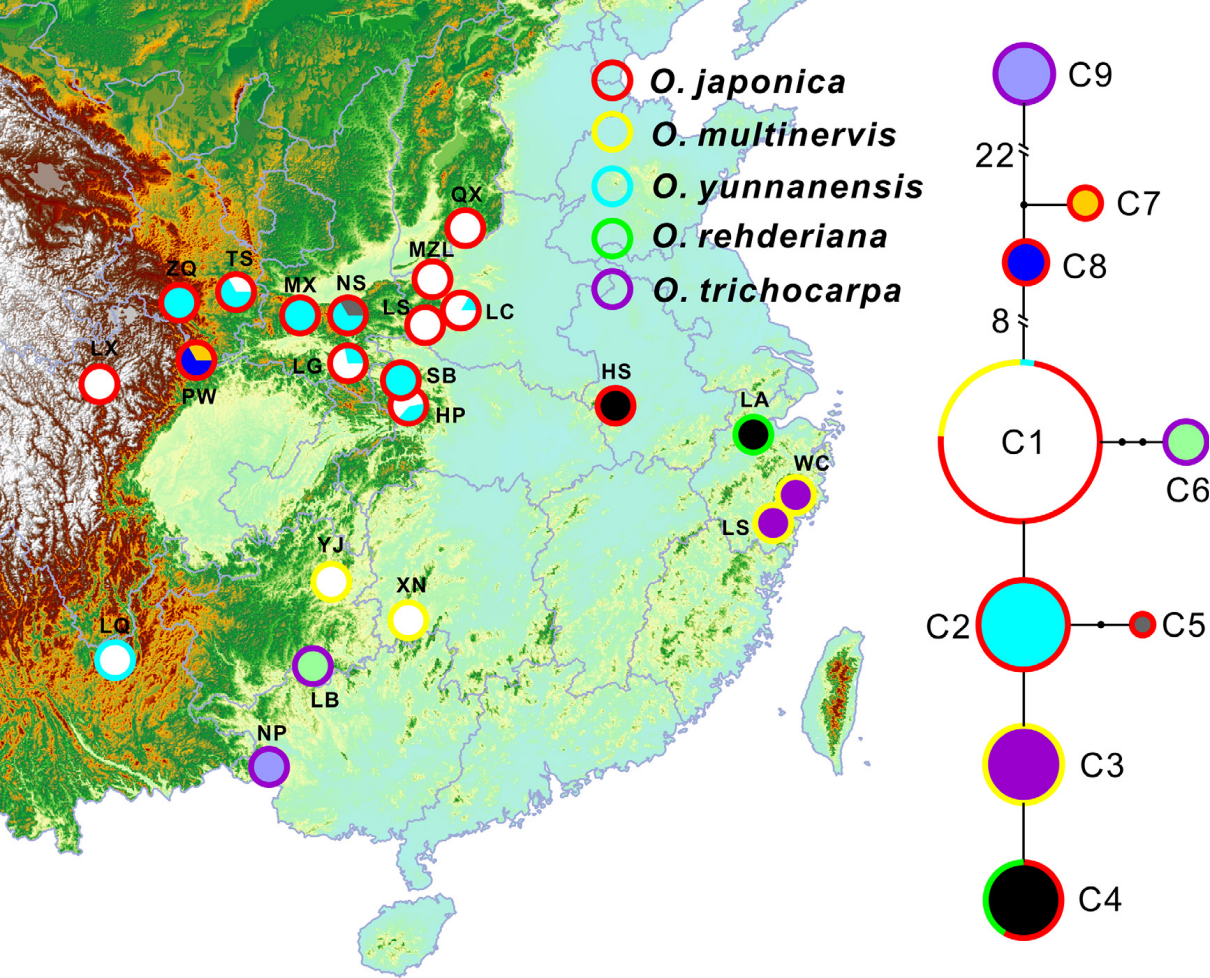
Distribution and network of the recovered haplotypes from five extant species.

The aligned sequences from four cpDNA fragments of 112 individuals are 2742 bp in length (Fig. S1; Table S2). According to the sequence variations, we recovered nine haplotypes (Tables 1 and 2). All haplotypes together clustered into three groups (Fig. 1). The first one comprised C1, C2, C3, C4, C5, and C6. C1 was found for *O. japonica*,* O. multinervis,* and *O. yunnanensis* while C4 for *O. japonica* and *O. rehderiana*. C2 and C5 were found for *O. japonica*. C3 was fixed for *O. multinervis* while C6 for *O. trichocarpa*. The second group comprised C7 and C8 found for *O. japonica*. The last one comprising C9 was only detected for *O. trichocarpa*.

**Table 2:** Variable positions of cpDNA fragments were detected in Chinese *Ostrya* species

	Variable positions																															
	*trnL*–*trnF* (944 bp)														*psbA*–*trnH* (451 bp)						*rps16* (797 bp)										*trnG* (547 bp)	
Haplotypes	7	1	2	5	5	5	5	9	4	7	7	8	8	8	1	1	1	1	2	3	8	9	2	3	3	3	4	5	5	6	9	4
	4	3	8	2	5	5	6		1	2	5	1	1	6	0	2	3	4	2	1	1	7	6	8	9	9	0	1	3	6		1
		2	2	0	1	7	5		4	0	6	5	6	7	3	2	0	0	8	6			5	7	6	7	1	5	4	6		4
C1	▲	G	A	G	–	G	C	T	C	A	T	T	–	T	G	♦	T	–	C	A	G	T	T	A	T	T	–	T	–	C	T	C
C2	▲	G	A	G	–	G	C	T	C	A	T	T	–	–	G	♦	T	–	C	A	G	T	T	A	T	T	–	T	–	C	T	C
C3	▲	G	A	G	–	G	C	T	C	A	T	T	T	–	G	♦	T	–	C	A	G	T	T	A	T	T	–	T	–	C	T	C
C4	▲	G	A	G	–	G	C	T	C	A	T	T	T	–	T	♦	T	–	C	A	G	T	T	A	T	T	–	T	–	C	T	C
C5	▲	G	A	G	–	G	C	G	A	A	T	T	–	–	G	♦	T	–	C	A	G	T	T	A	T	T	–	T	–	C	G	A
C6	▲	G	A	G	–	G	C	T	C	A	T	T	–	T	G	♦	A	–	T	A	G	T	T	A	T	T	–	T	▼	C	T	C
C7	–	G	A	G	–	C	C	G	A	A	T	T	–	T	T	?	T	–	C	T	T	T	T	G	T	T	–	G	–	A	G	A
C8	–	G	A	G	–	C	C	T	C	A	T	T	–	T	T	?	T	–	C	T	T	T	T	G	T	T	–	G	–	A	T	C
C9	–	A	C	A	A	G	A	G	C	G	G	–	–	–	G	♦	T	A	C	A	G	–	A	G	A	A	A	G	–	C	G	C

“▲” indicates “AAATT”; “▼” indicates “ATATAA”; “♦” indicates “TTTCATTT”; “■” indicates “ATAT”; “–” indicates a deletion; and “?” indicates the region of poly‐A/T structure.

### ITS sequence variations and phylogenetic analyses

**Figure 2:**
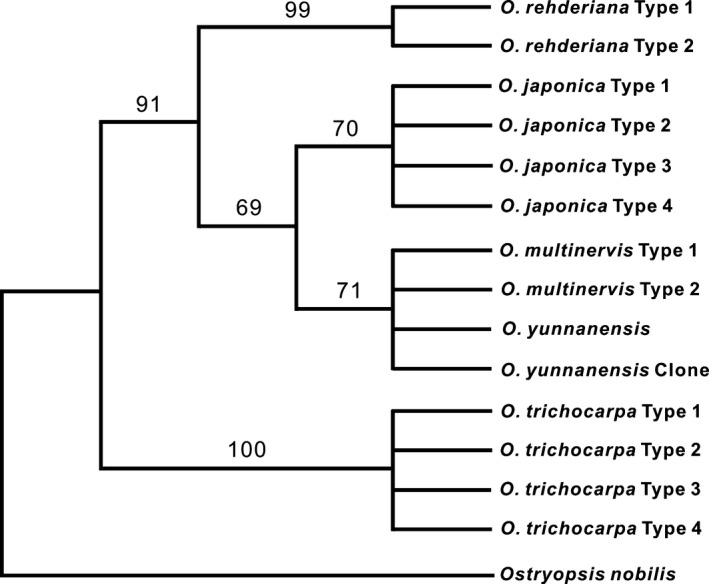
Most parsimonious tree constructed from internal transcribed spacer sequences to show the phylogenetic relationship of all species in China.

The aligned ITS sequences were 631 bp in length, and 40 nucleotide or indel variations were recovered across all sampled individuals of five species (Table 3). *O. trichocarpa* possessed 23 specific variations while *O. rehderiana* had seven ones. However, both species had two additive sites. For *O. japonica* and *O. multinervis*, only one specific variation for each of them was detected (Table 3). Sequences of the fresh leaves and specimen leaf fragments of *O. yunnanensis* are the same as those of *O. multinervis*. Phylogenetic analyses of all ITS sequences identified four well‐discerned lineages comprising *O. trichocarpa, O. rehderiana*,* O. japonica,* and *O. multinervis*–*O. yunnanensis* (Fig. 2). Further analyses included them and three species from Europe and North America (Fig. S2). Three Chinese species and *O. carpinifolia* from Europe comprised a Eurasian clade. The second clade contained *O. virginiana* and *O. konwltonii* from North America while the final clade contained only *O. trichocarpa* endemic to China.

**Table 3:** Nucleotide sites showing variation between the major internal transcribed spacer sequences identified in the present study

	Variable positions																																							
Species	5	5	6	7	1	1	1	1	1	1	1	1	1	2	2	2	2	2	2	2	2	4	4	4	4	4	4	5	5	5	5	5	5	5	5	5	5	5	5	5	
	7	8	6	8	3	5	5	5	7	8	9	9	9	0	0	0	1	2	2	3	6	1	2	3	4	4	5	0	2	3	3	4	5	5	6	6	6	7	7	8	
					5	2	6	8	8	5	0	1	3	3	6	8	4	1	9	3	5	1	5	1	3	4	2	8	2	0	1	0	2	9	1	2	9	5	8	8	
*O. japonica Type1*	C	G	A	C	C	A	G	A	C	–	G	C	–	T	T	G	C	G	–	Y	A	A	G	G	G	G	G	G	G	C	T	A	R	C	T	▲	A	A	A	A	
*O. japonica Type2*	C	G	A	C	C	A	G	A	C	–	G	C	–	T	T	G	C	G	–	C	A	A	G	G	G	G	G	G	G	C	T	A	G	C	T	▲	A	A	A	A	
*O. japonica Type3*	C	G	A	C	C	A	G	A	C	–	G	C	–	T	T	G	C	G	–	Y	A	A	G	G	G	G	G	G	G	C	T	A	G	C	T	▲	A	A	A	A	
*O. japonica Type4*	C	G	A	C	C	A	G	A	C	–	G	C	–	T	T	G	C	G	–	T	A	A	G	G	G	G	G	G	G	C	T	A	G	C	T	▲	A	A	A	A	
*O. multinervis Type1*	C	G	A	C	C	A	G	A	C	–	G	C	–	T	T	G	C	G	–	C	A	A	G	R	R	G	G	G	G	C	T	A	G	C	C	▲	G	G	A	A	
*O. multinervis Type2*	C	G	A	C	C	A	G	A	C	–	G	C	–	T	T	G	C	G	–	C	A	A	G	A	A	G	G	G	G	C	T	A	G	C	C	▲	G	G	A	A	
*O. yunnanensis* Clone	C	G	A	C	C	A	G	A	C	–	G	C	–	T	T	G	C	G	–	C	A	A	G	A	A	G	G	G	G	C	T	A	G	C	C	▲	G	G	A	A	
*O. yunnanensis*	C	G	A	C	C	A	G	A	C	–	G	C	–	T	T	G	C	G	–	C	A	A	G	G	A	K	G	G	G	C	T	A	G	C	Y	▲	G	G	A	A	
*O. rehderian Type1*	C	G	A	C	C	G	G	A	C	–	A	C	–	T	C	G	T	A	–	C	A	C	A	G	G	G	A	G	G	C	T	A	G	C	C	▲	G	A	A	A	
*O. rehderian Type2*	C	G	A	C	C	G	G	A	C	–	A	C	–	T	C	G	T	A	–	C	A	M	A	R	G	G	A	G	G	C	T	A	G	C	C	▲	G	A	A	A	
*O. trichocarpa Type1*	T	C	G	T	T	A	T	T	T	C	A	T	T	C	C	A	C	C	G	C	G	A	A	G	A	G	G	A	A	C	C	G	G	T	C	–	A	A	T	G	
*O. trichocarpa Type2*	T	C	G	C	T	A	T	T	T	C	A	T	T	C	C	A	C	C	G	C	G	A	A	G	A	G	G	A	A	C	C	G	G	T	C	–	A	A	T	G	
*O. trichocarpa Type3*	T	C	G	Y	T	A	T	T	T	C	A	T	T	C	C	A	C	C	G	C	G	A	A	G	A	G	G	A	A	C	C	G	G	T	C	–	A	A	T	G	
*O. trichocarpa Type4*	T	C	G	C	T	A	T	T	T	C	A	T	T	C	C	A	C	C	G	C	G	A	A	G	A	G	G	A	A	Y	C	G	G	T	C	–	A	A	T	G	

“▲” indicates “GCCC” and “–” indicates a deletion.

### Morphological clustering based on the morphological traits

**Figure 3:**
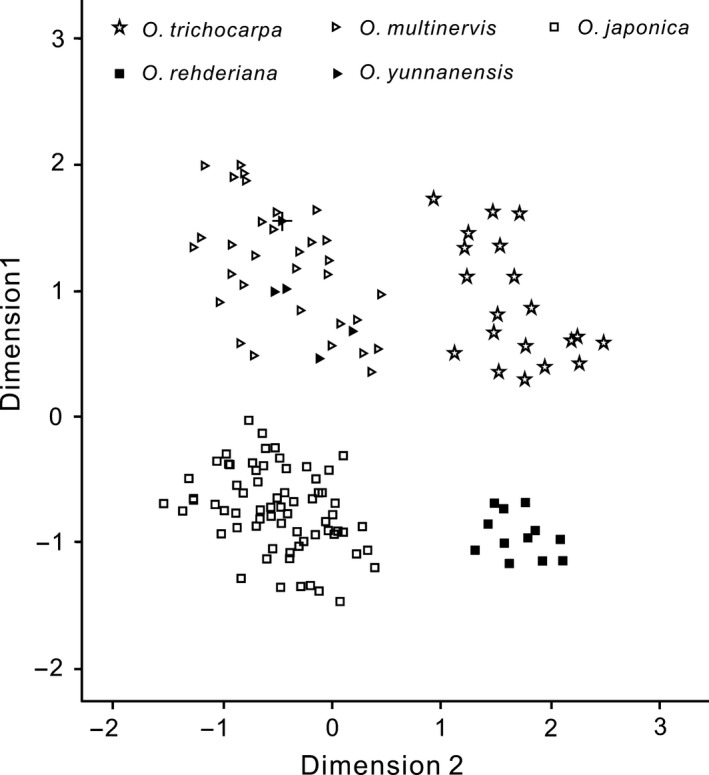
Morphological clustering based on the principal coordinate analysis. The black triangle with cross represents the type specimen of *Ostrya yunnanensis*.

Four groups were delimited based on morphological statistic analyses (Fig. 3). Dimension 1 (38.89%) divided all samples into two clusters. One comprised the individuals ascribed to *O. trichocarpa*,* O. yunnanensis,* and *O. multinervis* while the other contained those to *O. rehderiana* and *O. japonica*. Dimension 2 (22.19%) also divided these sampled individuals into two clusters. One consisted of those ascribed to *O. rehderiana* and *O. trichocarpa,* and the other comprised the remaining individuals. Totally, morphological clustering discerned four groups through combining dimension 1 and dimension 2, corresponding to *O. rehderiana*,* O. trichocarpa*,* O. japonica,* and *O. multinervis* while *O. yunnanensis* nested within *O. multinervis*.

## Discussion

It is widely acknowledged that genetic gaps should be integrated into species delimitation and taxonomic revision (Schlick‐Steiner et al. 2010; Fujita et al. 2012; Carstens et al. 2013; Hu et al. 2015; Su et al. 2015). In this study, we aimed to clarify species boundaries of Chinese hop‐hornbeams according to the collected materials across the distributional ranges of each species. Our results based on molecular and morphological evidences suggested a consistent result that the only four of five species should be maintained in the future while one species (*O. yunnanensis*) is conspecific to *O. multinervis*. Our further phylogenetic analyses including more species occurring out of China suggested that *O. trichocaipa* comprises a distinctive lineage different from congeners. In addition, sequence variations from cpDNA fragments failed to delimitate Chinese hop‐hornbeams species because of the incomplete lineage sorting and/or introgression in the history. However, our results suggested that sequence variations from the nuclear ITS region are effective to delimitate and discern the closely related species.

### Species delimitation and interspecific relationship

ITS sequence variations and phylogenetic analyses identified four well‐delimitated clusters (Fig. 2), corresponding to four extant species, *O. japonica*,* O. multinervis, O. rehderiana,* and *O. trichocarpa*. These four species are also discerned by morphological traits (Fig. 3). *O. multinervis* and *O. japonica* are closely related to each other with the limited number of ITS mutations while *O. rehderiana* is distant from these two species with more species‐specific ITS mutations (Table 3). Another interesting finding of the present study is that all ITS sequences recovered from *O. yunnanensis* based on fresh materials collected from the field and type specimen are similar to those of *O. multinervis*. Phylogenetic analyses undoubtedly placed them within *O. multinervis*. In addition, chloroplast haplotype of *O. yunnanensis* was also found for *O. multinervis*. We also examined morphological distinctions between *O. yunnanensis* and *O. multinervis* and found that no trait can differentiate them completely. All of these lines of evidence obviously suggested that *O. yunnanensis* should be reduced to *O. multinervis* (Wu 1991).

Taxonomic and phylogenetic position of *O. trichocarpa* remains unclear because few specimens and materials are available (Li and Skvortsov 1999; Yoo and Wen 2002, 2007; Li 2008; Grimm and Renner 2013). Our results clearly suggested that this species is well delimitated from the remaining three species occurring in China based on both morphological and molecular evidence. In addition, our phylogenetic analyses containing more species from Europe and North America suggested that *O. trichocarpa* comprised an independent lineage while the other three Chinese species clustered into a Eurasian lineage with one European species *O. carpinifolia* (Yoo and Wen 2002, 2007). Two species from North America clustered into a third lineage. All of our results suggested that interspecific relationships in *Ostrya* need further examinations based on more evidence.

### Discrimination powers of ITS and cpDNA sequence variations

Our results suggested that discrimination powers of ITS and cpDNA sequence variations are different (Figs. 1, 2, and S1). Although the total length of ITS regions (631 bp) is extremely smaller than four cpDNA fragments (2742 bp), the detected numbers of mutations of ITS sequences (40 mutations) were significantly more than cpDNA fragments (32 mutations) (Tables 2 and 3). ITS variations could discern four well‐delimitated species in *Ostrya*. However, sequence variations of a single or four cpDNA fragments together failed to identify these four species (Fig. S1). Numerous studies suggested that sequence variations from cpDNA fragments could not discern closely related species (Starr et al. 2009; Wang et al. 2011a; Hassel et al. 2013) while the use of nrITS seems to be more effective in delimitating closely related species (Li et al. 2011). Several factors, including slow rates of cpDNA evolution, interspecific introgression as well as incomplete lineage sorting may account for the low discriminatory power of cpDNA markers (Hollingsworth et al. 2011). Hybridization and introgression are common in plants (Abbott et al. 2010). Our results suggested that some haplotypes were shared by two species (Fig. 1), and the recovered haplotypes clustered into three tentative clades (Fig. S1). One clade comprising two haplotypes (C7 and C8) were fixed only for *O. japonica,* and another clade comprising C9 for *O. trichocarpa*. Both species contained two highly divergent clades, possibly suggesting that one clade might have been introgrossed from other species if the population size of each species is not large enough to comprise highly divergent haplotypes. A few additive sites were also detected for ITS may also suggest possible hybridizations in the history (Álvarez and Wendel 2003; Mallet 2007; Wang et al. 2009). However, all of four species shared the closely related haplotypes in one clade (C1 to C6), suggesting possible incomplete lineage sorting between these haplotypes. Therefore, hybridization and incomplete lineage sorting may have together resulted in the low discrimination of the cpDNA sequence variations.

However, the high success of nrITS in discriminating closely related species may possibly be ascribed to its fast rate of mutation and lineage sorting in angiosperms (Wang et al. 2011b). It should be noted that the contrasting scenarios probably co‐occur in the sister genera (Hu et al. 2015). In one genus, nrITS was highly effective in discriminating closely related species, but cpDNA failed; however, in the other genus, the discriminatory ability of the nrITS sequence variations was noticeably lower than that of the cpDNAs in delimiting two species of this genus. Therefore, nrITS and cpDNA regions should be together used for delimitating closely related species in the practice studies in the future. In addition, more samples across the distributional range of each species should be used for such studies.

## Conflict of Interest

None declared.

## Supporting information


**Figure S1.** Network analysis of cpDNA for each fragment and coalescent fragments. Gaps were treated as a fifth character state, considering neighboring gaps as single events. Circle size is proportional to haplotypes frequencies. Locus names and corresponding length are shown above each network. Different dot color indicates the different taxa. Red: *O. japonica*. Yellow: *O. multinervis*. Cyan: *O. yunnanensis*. Green: *O. rehderiana*. Purple: *O. trichocarpa*.

**Figure S2.** Most parsimonious tree of Chinese hop‐hornbeams and three species from Europe and North America based on ITS sequences. Different color indicates *Ostrya* species in the different regions. Red: China. Blue: Europe. Black: North America.

**Table S1.** The primer pairs used in this study.
**Table S2.** Morphological characters were reexamined in Chinese *Ostrya*.

**Appendix S1.** Taxonomic treatments.
