# Supplementary figures and images for: Species delimitation of Chinese hop‐hornbeams based on molecular and morphological evidence
Source: Ecol Evol. 2016 Jun 13;6(14):4731–40. doi: 10.1002/ece3.2251 (PMC4979702; doi:10.1002/ece3.2251)

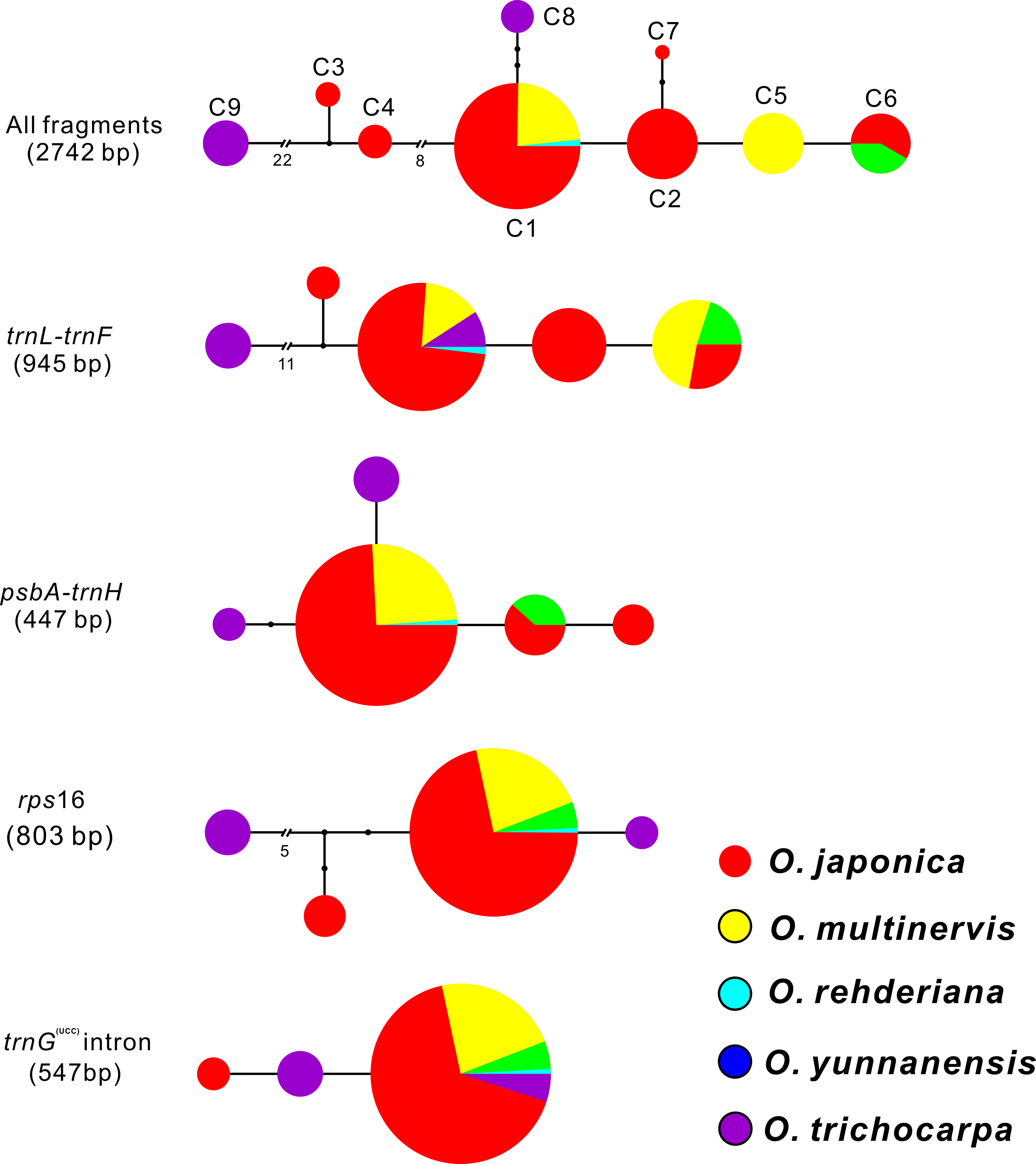

Supplement: Supplementary file 1 — Figure S1. Network analysis of cpDNA for each fragment and coalescent fragments. Gaps were treated as a fifth character state, considering neighboring gaps as single events. Circle size is proportional to haplotypes frequencies. Locus names and corresponding length are shown above each network. Different dot color indicates the different taxa. Red: O. japonica. Yellow: O. multinervis. Cyan: O. yunnanensis. Green: O. rehderiana. Purple: O. trichocarpa. [file ECE3-6-4731-s001.tif]

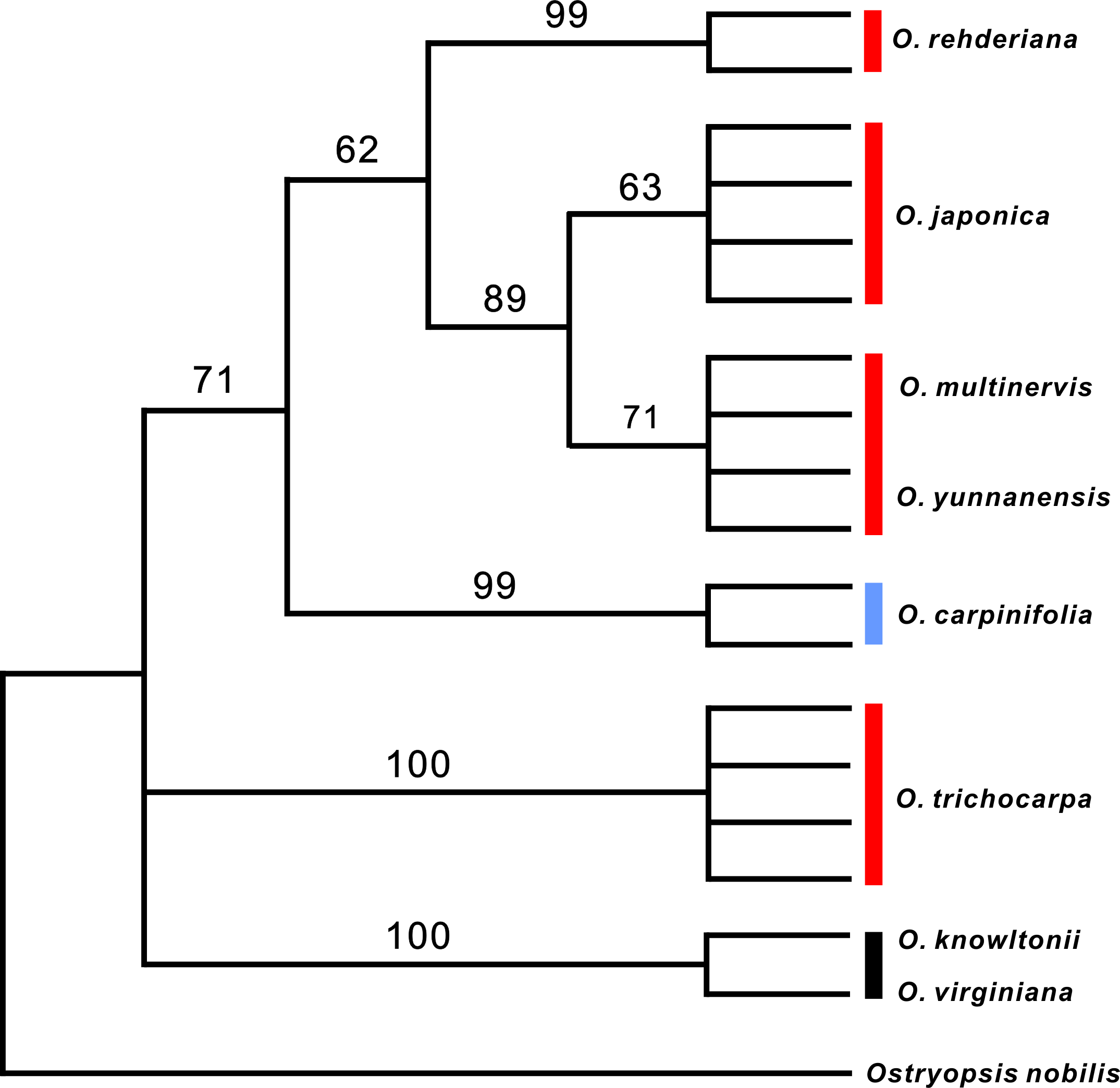

Supplement: Supplementary file 2 — Figure S2. Most parsimonious tree of Chinese hop‐hornbeams and three species from Europe and North America based on ITS sequences. Different color indicates Ostrya species in the different regions. Red: China. Blue: Europe. Black: North America. [file ECE3-6-4731-s002.tif]
