# Supplementary material for: Species delimitation of Chinese hop‐hornbeams based on molecular and morphological evidence
Source: Ecol Evol. 2016 Jun 13;6(14):4731–40. doi: 10.1002/ece3.2251 (PMC4979702; doi:10.1002/ece3.2251)
Supplement: Supplementary file 3 — Table S1. The primer pairs used in this study. Table S2. Morphological characters were reexamined in Chinese Ostrya. [file ECE3-6-4731-s003.doc]

**Tables S1** The premier pairs used in this study.

| Locus | Length (bp) | Primer pairs (5'-3') | | References |
| --- | --- | --- | --- | --- |
| *psbA-trnH* | 446 | *psbA* | GTTATGCATGAACGTAATGCTC | Sang et al. 1997 |
|  |  | *trnH* | CGCGCATGGTGGATTCACAAATC | Sang et al. 1997 |
| ITS | 631 | ITS1a | TCCTCCGCTTATTGATATGC | White et al. 1990 |
|  |  | ITS4a | GGAAGTAAAAGTCGTAACAAGG | White et al. 1990 |
| *rbcL* | 1096 | *rbcL*-F | ATGTCACCACAAACAGAGACT | Liu et al. 2014 |
|  |  | *rbcL*-R | CCGAATTGTAGTACGGAATC | Liu et al. 2014 |
| *matK* | 843 | *matK*1R | ACCCAGTCCATCTGGAAATCTTGGTTC | CBOL Plant Working Group 2009 |
|  |  | *matK*3F | CGTACAGTACTTTTGTGTTTACGAG | CBOL Plant Working Group 2009 |
| *trnV-trnM* | 885 | *trnV* | GCTATACGGGCTCGAACC | Cheng et al. 2005 |
|  |  | *trnM* | TACCTACTATTGGATTTGAACC | Cheng et al. 2005 |
| *trnL- trnF* | 945 | *trnL*(UAA)5'exon | CGGAATCGGTAGACGCTACG | Taberlet et al. 1991 |
|  |  | *trnF*(GAA) | ATTTGAACTGGTGACACGAG | Taberlet et al. 1991 |
|  |  | *TrnL* | AAAATCGTGAGGGTTCAAGTC | Sang et al. 1997 |
|  |  | *TrnF* | GATTTGAACTGGTGACACGAG | Sang et al. 1997 |
| *rps16* | 803 | *rps*F | GTGGTAGAAAGCAACGTGCGACTT | Oxelman et al. 1997 |
|  |  | *rps*MR2 | GGTTTAGACATTACTTCGTTGA | Oxelman et al. 1997 |
| *trnG* | 547 | *trnG* F | GGTAAAAGTGTGATTCGTTC | Nishizawa and Watano 2000 |
|  |  | *trnG* R | GTTTCATTCGGCTCCTTTAT | Nishizawa and Watano 2000 |

**Table S2** Morphological characters were reexamined at the population level in Chinese *Ostrya*. The unbold characters were found merely within species and not used.

| Characters number | State | Type | coding |
| --- | --- | --- | --- |
| Leaf characters |  |  |  |
| 1 | a: Longest length (cm) | quantitative | 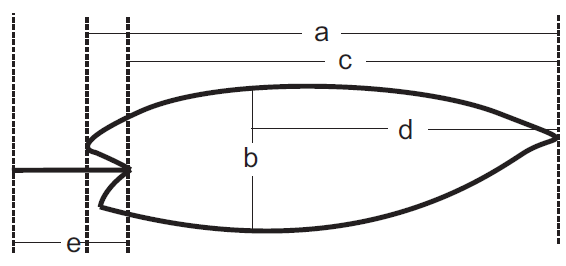 |
| 2 | b: The widest width (cm) | quantitative |  |
| 3 | c: Shortest length (cm) | quantitative |  |
| 4 | d (The widest width started to measure from the base): Vertical distance from the first to reach the widest  place to the tip of leaf (cm) | quantitative |  |
| **5** | **e: Length of petiole (mm)** | **quantitative** |  |
| **6** | **f:** **Number of lateral veins on each side of midvein** | **quantitative** | - |
| **7** | **a/b** | **quantitative** |  |
| 8 | a/f | quantitative |  |
| 9 | b/f | quantitative |  |
| 10 | a-d | quantitative |  |
| **11** | **a-c** | **quantitative** |  |
| 12 | c/e | quantitative |  |
| **13** | **a/e** | **quantitative** |  |
| **14** | **d/b** | **quantitative** |  |
| **15** | **d/(a-d)** | **quantitative** |  |
| 16 | (a-d)/b | quantitative |  |
| 17 | Distance between the sixth  and seventh lateral veins (mm) | quantitative |  |
| 18 | Densely pubescent or not on the leaf | qualitative | Densely pubescent: 1 |
|  |  |  | Not densely pubescent: 2 |
| **19** | **Densely pubescent or not on the petiole** | **qualitative** | **Densely pubescent: 1** |
|  |  |  | **Not densely pubescent: 2** |
| **Nutlet** |  |  |  |
| **20** | **Glabrous or densely pubescent** | **qualitative** | **Glabrous: 1** |
|  |  |  | **Densely pubescent: 2** |
| **Bract** |  |  |  |
| **21** | **Constricted into a stipe at base or not** | **qualitative** | **Not constricted into a stipe at base: 1** |
|  |  |  | **Constricted into a stipe at base: 2** |

**References**

Cheng, Y. P., S. Y. Hwang, and T. P. Lin, 2005. Potential refugia in Taiwan revealed by the phylogeographical study of *Castanopsis carlesii* Hayata (Fagaceae). Mol. Ecol. 14(7):2075-2085

Liu, B. B., R. J. Abbott, Z. Q. Lu, B. Tian, and J. Q. Liu. 2014. Diploid hybrid origin of *Ostryopsis intermedia* (Betulaceae) in the Qinghai-Tibet Plateau triggered by Quaternary climate change. Mol. Ecol. 23(12):3013-3027.

Nishizawa, T., and Y. Watano. 2000. Primer pairs suitable for PCR-SSCP analysis of chloroplast DNA in angiosperms. J. Phytogeogr. Taxon 48:63-66.

Oxelman, B., M. Lidén, and D. Berglund. 1997. Chloroplast *rps16* intron phylogeny of the tribe Sileneae (Caryophyllaceae). Pl. Syst. Evol. 206(1-4):393-410.

Sang, T., D. Crawford and T. Stuessy. 1997. Chloroplast DNA phylogeny, reticulate evolution, and biogeography of *Paeonia* (Paeoniaceae). Am. J. Bot. 84(8):1120-1120.

Taberlet, P., L. Gielly, G. Pautou, and J. Bouvet. 1991. Universal primers for amplification of three non-coding regions of chloroplast DNA. Pl. Mol. Biol. 17(5):1105-1109.

White, T. J., T. Bruns, S. Lee, and J. Taylor. 1990. Amplification and direct sequencing of fungal ribosomal RNA genes for phylogenetics. PCR protocols: a guide to methods and applications 18:315-322.

CBOL Plant Working Group. 2009. A DNA barcode for land plants. Proc. Natl Acad. Sci. USA 106(31):12794-12797.
