# Supplementary material for: Species delimitation of Chinese hop‐hornbeams based on molecular and morphological evidence
Source: Ecol Evol. 2016 Jun 13;6(14):4731–40. doi: 10.1002/ece3.2251 (PMC4979702; doi:10.1002/ece3.2251)
Supplement: Supplementary file 4 — Appendix S1. Taxonomic treatments. [file ECE3-6-4731-s004.doc]

Appendix

**Taxonomic treatments**

Our analyses based on both molecular and morphological evidence showed that *O. yunnanensis* is conspecific to *O. multinervis*. *O. multinervis* was described before *O. yunnanensis*. Therefore, *O. yunnanensis* should be considered as a synonym of *O.* *multinervis*.

*Ostrya multiveris* Rehder ≡ *Ostrya yunnanensis* Hu ex P. C. Li, Acta Phytotax. Sin. 17(1): 87. 1979, **syn. nov.** – Type: China, Yunnan, Luquan, 1952-12-05, P.Y. Mao 01935 (GUCAS).

**A new key to *Ostrya* species in China**

1. Bracts narrowly elliptic or obovate-lanceolate, 20–26 mm, constricted into a stipe at base; female inflorescence loosely racemose........................................*O. rehderiana*

1. Bracts elliptic, ovate-oblong, or obovate-oblong, 15–25 mm, not constricted into a stipe at base; female inflorescence densely racemose

2. Leaves with 10–17 lateral veins on each side of midvein and length–width ratio1.9–2.5, petiole glabrescent or pubescent

..............................................................................................................*O. japonica*

2. Leaves with 16–28 lateral veins on each side of midvein, length–width ratio 2.2–3.6, petiole pubescent

3. Nutlet glabrous; ribbed, petiole 5–10 mm, lateral veins not impressed adaxially........................................................................................*O. multinervis*

3. Nutlet densely pubescent; obscurely ribbed, petiole 2–5 mm, lateral veins impressed adaxially........................................……………........*O. trichocarpa*
